# A Novel HPLC Method for the Concurrent Analysis and Quantitation of Seven Water-Soluble Vitamins in Biological Fluids (Plasma and Urine): A Validation Study and Application

**DOI:** 10.1100/2012/359721

**Published:** 2012-03-12

**Authors:** Margherita Grotzkyj Giorgi, Kevin Howland, Colin Martin, Adrian B. Bonner

**Affiliations:** ^1^Centre for Health Services Studies, University of Kent, Canterbury, Kent CT2 7NF, UK; ^2^Department of Biosciences, University of Kent, Canterbury, Kent CT2 7PD, UK; ^3^School of Health, Nursing and Midwifery, University of the West of Scotland, Ayr Campus, Ayr KA8 0SX, Scotland, UK

## Abstract

An HPLC method was developed and validated for the concurrent detection and quantitation of seven water-soluble vitamins (C, B_1_, B_2_, B_5_, B_6_, B_9_, B_12_) in biological matrices (plasma and urine). Separation was achieved at 30°C on a reversed-phase C18-A column using combined isocratic and linear gradient elution with a mobile phase consisting of 0.01% TFA aqueous and 100% methanol. Total run time was 35 minutes. Detection was performed with diode array set at 280 nm. Each vitamin was quantitatively determined at its maximum wavelength. Spectral comparison was used for peak identification in real samples (24 plasma and urine samples from abstinent alcohol-dependent males). Interday and intraday precision were <4% and <7%, respectively, for all vitamins. Recovery percentages ranged from 93% to 100%.

## 1. Introduction

Water-soluble vitamins include B group vitamins (B_1_, B_2_, B_3_, B_5_, B_6_, B_9_, B_12_) and ascorbic acid (vitamin C). Vitamins are micronutrients that are essential to life, and many of them play an important role in regulation of brain functioning (e.g., vitamin B_1_ deficiency causes biochemical brain lesions that causes Wernicke's encephalopathy, an acute neuropsychiatric condition often observed in chronic alcohol abusers [3]).

The reference methods for vitamins analysis in biological fluids are often based on time-consuming microbiological assays that may lack specificity [4]. In addition, vitamin extraction involves pretreatment through complex chemical reactions followed by individual methods for the determination of each vitamin. During the last decades, there has been an increasing interest for the simultaneous determination of vitamins. Thus, various analytical methods have been developed over recent years [5–9]. A number of recent studies have focused on validation of analytical methodologies for multivitamins analysis but the vast majority of them applied their methods to analysis of food matrices, drinks, polyvitaminated premixes, and vitamins supplements [10, 11]. On the other hand, only a relatively small number of experimental studies focused on validation of analytical methodologies for multivitamins analysis in biological samples (blood and urine) and with limited results in terms of lengthy sample preparation steps and method's robustness and reproducibility [8]. Because of this lack of a robust and validated analytical test for multivitamin analysis in biological samples in routine clinical assessment and in those investigations where a timely and robust analytical method is needed, the aim of this study was to develop and validate a novel HPLC methodology for rapid detection and quantitation of seven water-soluble vitamins (B_1_, B_2_, B_5_, B_6_, B_9_, B_12_, C) in biological fluids (plasma and urine). The validated method was then applied to quantify water-soluble vitamins in plasma and urine samples obtained from 24 abstinent alcohol-dependent males.

## 2. Materials and Methods

### 2.1. Standards and Reagents

Water-soluble vitamins employed in this study (B_1_, B_2_, B_3_, B_5_, B_6_ as pyridoxal phosphate [5′-PLP], B_9_, B_12_ and C) and the internal standard (theobromine) were purchased from Sigma-Aldrich, Gillingham, UK, and were of the highest grade of purity available (>95%). Methanol, ethanol (HPLC grade), and n-hexane were obtained from Fisher Scientific, Loughborough, UK. Trifluoroacetic acid (TFA) of protein chemistry grade (>99.5%) was purchased from Thermo Scientific, Fisher Scientific, Loughborough, UK. Ultra pure HPLC grade water (Maxima water purification system, USF Elga, High Wycombe, UK) was used throughout the entire protocol.

### 2.2. Chromatographic Conditions

An Agilent 1100 chromatographic system (Agilent Ltd., South Queensferry, UK) was used for the analysis and quantitation of vitamins in biological samples. The ChemStation software controlled the whole chromatographic system.

Vitamins were separated on a reversed-phase chromatographic column MetaChem Polaris C18-A (5 *μ*m, 250 mm × 2.1 mm i.d., Varian medical system Ltd, Crawley, UK) fitted with a precolumn (MetaGuard column, C18-A, Varian medical system Ltd, Crawley, UK) using combined isocratic and linear gradient elution with a mobile phase consisting of 0.01% TFA aqueous (pH 2.9, solvent A) and 100% methanol (solvent B). Linear gradient profile (A : B) started at 95 : 5 and it was kept constant for the first 4 minutes, then linearly decreased up to 2 : 98 during the next 6 minutes, then it was kept constant in the next 20 minutes and finally linearly increased up to 95 : 5 in the last 5 minutes of separation. Total run time was 35 minutes. This gradient was utilised for temperature studies; subsequently, the timing of gradient was modified to suit reduced analysis time. The flow rate was adjusted to 0.2 mL/min. Injection volume was 3 *μ*L. Column temperature was kept constant at 30°C. Detection was performed with a photodiode array detector monitoring the eluent at 280 nm; however, quantitation was performed at maximum wavelength for each vitamin as follows: 230 nm for ascorbic acid, 270 nm for thiamine, 265 nm for riboflavin, 256 nm for nicotinamide, 266 nm for pantothenic acid, 257 nm for pyridoxine, 280 nm for folic acid, and 230 nm for cyanocobalamin. Identification of resolved peaks in real samples was executed by comparing their spectra with those derived from aqueous standard solutions.

### 2.3. Standards Preparation

The aqueous stock solutions of water-soluble vitamins (B_1_, B_3_, B_5_, B_6_ as pyridoxal phosphate [5′-PLP], B_9_, B_12_, C) were prepared weekly by weighting 10 mg of each vitamin in a volumetric cylinder in 100 mL of ultrapure water (Maxima water, USF Elga, High Wycombe, UK) containing 0.01% of trifluoroacetic acid (TFA). Vitamin B_2_ was prepared by weighting 5 mg and subsequently added to the multivitamin solution (final concentration of riboflavin was 50 ng *μ*L^−1^). After brief agitation, solution was transferred by pouring into an amber-glass bottle for storage at +4°C. The final concentration of each vitamin was 100 ng *μ*L^−1^ (except vitamin B_2_ which was 50 ng *μ*L^−1^). Vitamin B_9_ solution was prepared by weighting 5 mg of powdered Vitamin B_9_ in a volumetric cylinder and dissolved in 100 mL of 1 M NaHCO_3_. All solutions were stored in a refrigerator in amber-glass bottles to protect vitamins from light-induced oxidation. Working standard solutions were prepared fresh daily from stock solutions. Final concentration of water-soluble vitamins standards ranged from 0.25 to 25 ng *μ*L^−1^ (six concentration levels). Theobromine (internal standard) was used at a concentration of 2 ng *μ*L^−1^.

### 2.4. Sample Preparation

Experiments were carried out to identify a sample preparation procedure that would allow simultaneous detection of seven water-soluble vitamins in biological samples. Aliquots of one spiked plasma sample (final concentration of each water-soluble vitamins: 20 ng *μ*L^−1^) were concomitantly processed following one of these three procedures: deproteinisation with 400 *μ*L pure acetonitrile followed by solid phase extraction. The solid phase extraction method was obtained from [8]. The second methodology involved liquid-liquid extraction method [600 *μ*L *n*-hexane + 150 *μ*L ethanol : methanol, 95 : 5, v/v] with no solid phase extraction. The third methodology involved deproteinisation with 600 *μ*L ethanol : methanol, 95 : 5, v/v followed by solid phase extraction procedure as per [8]. Experiments were run in triplicate.

The first methodology was performed following the procedure reported in [8] with the minor modification of using 400 *μ*L of pure acetonitrile in a 1 : 1 ratio (v/v) to fresh or freshly thawed plasma previously spiked with internal standard (theobromine, 2 ng *μ*L^−1^).

The second methodology was carried out by transferring to a glass analysis tube 400 *μ*L of fresh or freshly thawed plasma containing IS. Six hundred microliters of pure *n*-hexane were added and tubes were briefly vortex mixed. The addition of hexane was used to extract lipid-soluble matrix components that may interfere with vitamins analysis. Tubes were then centrifuged at 4,000 rpm for 5 minutes at 4°C. After centrifugation was complete, 150 *μ*L of ethanol : methanol (95 : 5, v/v) was added to the tubes and centrifuged at 23,000 rpm for 15 minutes at 4°C. The upper layer (organic phase) was discarded. The lower layer (aqueous phase) was collected and placed in a new, capped microcentrifuge tube. Tubes were placed in a SpeedVac instrument (Thermo Scientific, Fisher Scientific, Loughborough, UK) to dry. When the supernatant from the aqueous phase was completely dried, samples were resuspended in two HPLC vials containing 0.01% TFA in water. No solid phase extraction procedure was carried out prior to HPLC injection.

The third methodology was carried out by adding 600 *μ*L of ethanol : methanol (95 : 5, v/v) to 400 *μ*L of fresh or freshly thawed plasma. Samples were vortex-mixed briefly (30 seconds) and samples were centrifuged at 15,000 rpm for 15 minutes at 4°C. Supernatant was carefully collected and placed in a new capped microcentrifuge tube and placed in a SpeedVac instrument. When dry, samples were resuspended in water : methanol (50 : 50, v/v) before application to solid phase cartridge. Solid phase extraction was carried out following the procedure reported in [8].

The same procedures were also used for urine samples with no modification.

### 2.5. Solid-Phase Extraction Procedure

Solid-phase extraction (SPE) procedure was carried out following the protocol described by Chatzimichalakis and colleagues [8] with no modifications.

### 2.6. Method Development

#### 2.6.1. Temperature Studies

Temperature studies were carried out to evaluate the run temperature that provides the best peak resolution in least time. Five HPLC injections were performed from one standard solution of water-and lipid-soluble vitamins and internal standard and the experiment was run in triplicate for each selected temperature (20, 25, 30, and 40°C).

#### 2.6.2. System Suitability

The system suitability was evaluated by five replicate analyses of a standard aqueous mixture of water-soluble vitamins (20 ng *μ*L^−1^ each vitamin). The acceptance limit was ±5% for the percent coefficient of variation (%CV) of the peak area and the retention time of water-soluble vitamins.

#### 2.6.3. Linearity (Calibration Curve)

Three calibration curves were constructed on three consecutive days. Linearity was tested by running six standard mixtures of water-soluble vitamins, at final on-column concentrations of 0.5, 1, 2, 5, 10, 15, and 20 ng *μ*L^−1^. The internal standard, theobromine, was kept at a constant concentration of 2 ng *μ*L^−1^.

#### 2.6.4. Accuracy

Accuracy, defined as the nearness of the true value and found value, was evaluated as %bias for water-soluble vitamins according to the following equation:

(1) $$\%\text{accuracy}=\frac{\text{observed}\;\text{concentration}}{\text{nominal}\;\text{concentration}}\;\times 100.$$

#### 2.6.5. Specificity

The specificity of an analytical method may be defined as the ability to detect the analyte peak in the presence of all the matrix components. In this case, a standard aqueous solution of water-soluble vitamins and the internal standard at known concentration (20 ng *μ*L^−1^ and 2 ng *μ*L^−1^, resp.) were spiked in a matrix of simulated plasma (composition obtained from [12]). Simulated plasma samples were processed as real plasma samples.

#### 2.6.6. Precision

Precision of the assay was determined by repeatability and intermediate precision for 3 consecutive days. Four different concentrations of water-soluble vitamins (2, 5, 10, and 15 ng *μ*L^−1^) were analysed in five independent series during the same day (intraday precision) and over 3 consecutive days (intermediate precision). Every sample was injected three times.

#### 2.6.7. Limits of Detection and Quantitation (Sensitivity)

Limit of detection (LOD) and limit of quantitation (LOQ) were estimated from the signal-to-noise ratio. LOD is defined as the lowest concentration resulting in a peak area of three times the baseline noise. LOQ is defined as the lowest concentration that provides a signal-to-noise ratio higher than 10, with precision (%CV) and accuracy (%bias) within their acceptable range (10%).

#### 2.6.8. Stability

The stability of the water-soluble vitamins solution was determined by analyzing standard aqueous solutions and spiked, simulated plasma samples after a short-term storage at controlled room temperature (20–25°C) and at +4°C for 12 and 24 h. The long-term stability was determined by analysing samples stored at +4°C for 30 days. The autosampler stability was determined by analysing the samples after 24 h of storage in the autosampler (set at +4°C ± 2°C).

#### 2.6.9. Recovery Studies

The percentage recovery rate (% recovery) was calculated using the experimental response values and values provided by the calibration curves for the same quantity of analyte. Student's *t-*test was performed to assess whether the recovery rate was significantly different from 100% at *P* < 0.05.

### 2.7. Statistical Analysis

Data collected in this study were analysed using SPSS version 17 statistical package by one-way analysis of variance (ANOVA) and by independent-samples Student's *t-*test. Univariate linear regression analysis using least square method was applied to test the model. Correlation coefficient was calculated and the results of the statistical analysis were considered significant if their corresponding *P* values were less than 0.05.

## 3. Results

### 3.1. Temperature Study

A temperature study was conducted by analysing a standard mixture of water-soluble vitamins, at known concentration, using 4 column temperatures. Experiments were run in triplicate. Results are reported in Table 1. Column temperature of 30°C was selected as an acceptable compromise between rapid separation (35 minutes) and analytes degradation measured as decreased detector response.

### 3.2. System Suitability

Results from system suitability studies are reported in Table 2.

### 3.3. Plasma Sample Extraction Method Development

A series of experiments were carried out to identify a sample preparation procedure that would allow simultaneous detection of eight water-soluble vitamins in plasma. Aliquots of one plasma sample spiked with water-soluble vitamins, 20 ng *μ*L^−1^ each vitamin, were concomitantly processed following one of the three procedures detailed in Section 2. Experiments were run in triplicate. Results are reported in Table 3.

Results indicate that there was no statistically significant difference between the three sample preparation methods (Student's *t-*tests not significant, *P* > 0.05), except for 5′-PLP when extracted with the second method (liquid-liquid extraction) when compared with the third method (600 *μ*L ethanol : methanol, 95 : 5, v/v, followed by solid phase extraction): the difference between peak areas was statistically significant beyond 5% (Student's *t-*test, *P* < 0.05; exact *P* = 0.017). Specifically, 5′-PLP extraction was better with the second method in comparison to the third one (peak areas: 145.0723 mAU and 60.97128 mAU, resp.). Unfortunately, when spiked in plasma sample, vitamin B_3_ (nicotinamide) was not detectable by our method.

## 4. Method Validation

### 4.1. Specificity, Linearity, and Precision Studies

**Figure 1:**
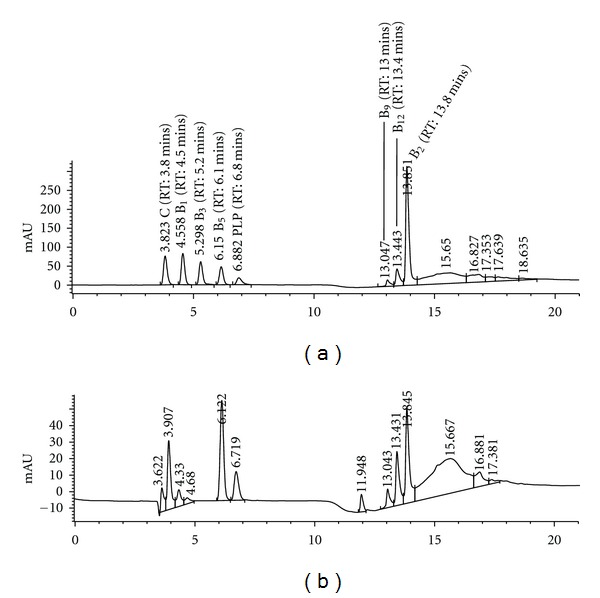
Vitamins mix (standards prepared in water, 20 ng/*μ*L) analysed under standardised conditions reported in the main text. (a) Real plasma sample obtained from one study participant analysed under standardised conditions reported in the main text. (b) Real plasma sample was spiked with the IS (RT: 11.948).

Representative chromatograms of standard solution and a real plasma sample are depicted in, respectively, Figures 1(a) and 1(b). Water-soluble vitamins elute in a specific order and in groups depending upon their chemical properties and interaction with the analytical column. As expected, polar vitamins (ascorbic acid, thiamin, nicotinamide and PLP) elute first, followed by pantothenic acid and finally by low-polar vitamins (folic acid, cyanocobalamin and riboflavin). Retention times were as following: 3.5 ± 0.03 mins [C], 4.3 ± 0.03 mins [B_1_], 5.3 ± 0.06 mins [B_3_], 6.1 ± 0.06 mins [5′-PLP], 6.8 ± 0.02 mins [B_5_], 12 ± 0.02 mins [theobromine, IS], 13 ± 0.01 mins [B_9_], 13.4 ± 0.08 mins [B_12_], and 13.8 ± 0.03 mins [B_2_]. As reported in Figure 1, peaks are well resolved and symmetric. Peak identification and purity were investigated by comparing UV spectra of each individual vitamin when analysed in mixtures and by running standard samples containing only one vitamin. When comparing standard chromatogram with chromatograms obtained from spiked artificial plasma, no major interference was noted from endogenous substances naturally present in human plasma. Two unidentified peaks were noted in spiked sample chromatogram; however, they did not interfere with vitamins quantitation.

Linearity was tested by running six standard mixtures of water-soluble vitamins at final, on-column concentrations of 0.5, 1, 2, 5, 10, 15, and 20 ng *μ*L^−1^. The method was linear across the whole range of concentrations (Table 4).

For all peaks, there was a very tight relationship between the amount of vitamins and the detectors response as indicated by *R* ^2^ values that exceeded 0.996 (±0.002).

Linearity of the method was also investigated in spiked plasma samples (data not shown). No statistically significant difference was observed between slopes of regression lines generated by aqueous- and plasma-based standards (Student's *t-*test not significant, *P* > 0.05). So for quantitation purposes, aqueous-based standards were used to calibrate the instrument response when analysing trial samples. Calibration curves were constructed using six standard concentrations of eight water-soluble vitamins prepared in double distilled water, and they were run in triplicate. For each curve, peak-areas of vitamins were plotted against the nominal (theoretical) vitamins concentration. Calibration curves were generated by weighted (1/*y*) linear regression analysis.

Detection/quantitation limits were determined by analysis of six standard solutions and three spiked plasma samples with final concentrations ranging from 0.5 ng *μ*L^−1^ to 30 ng *μ*L^−1^ each vitamin.

Precision of the method was evaluated by estimating the repeatability and intermediate precision of the analytical method. The repeatability was studied by running 10 consecutive replications of the same sample and calculating the %RSD for peaks area and elution times (sample injection volume = 3 *μ*L). The intermediate precision was calculated as the %RSD of peaks area and elution times across three consecutive analytical days.

Values of %RSD for retention times and peak areas obtained in the analysis of reproducibility and intermediate precision are presented in Table 5.

All RSD values were similar to those reported in the literature for within- and between-days variation [7, 10]. Results indicate that repeatability and the intermediate precision of the method were acceptable.

### 4.2. Stability, Carryover, and Recovery

Stability of vitamins stock solutions was tested by analysing aliquots stored at different temperatures (room temperature [20°C], +4°C, −20°C, and −80°C) at different time intervals (same day of preparation, 7 days, 14 days and 30 days). Aliquots were stored either in clear autosampler vials or amber glass autosampler vials to investigate the effect of photodegradation on vitamins. Experiments were run in triplicate and average peak area (±RSD) was considered in calculations.

Results indicate that there is no statistically significant difference between standard solutions stored at −20°C and −80°C at every time point (Student's *t*-test not significant, *P* > 0.05); there is no statistically significant difference between standards kept at room temperature and +4°C on the same day of preparation up to 14 days (*P* > 0.05), whereas a statistical significant difference can be observed after 30 days (*P* < 0.05; exact *P* = 0.029). There is a statistical significant difference between standards kept at +4°C and −20°C after 30 days (*P* < 0.05; exact *P* = 0.031) but no difference was noted between standards analysed on the same day of preparation, and after 7 and 14 days (*P* > 0.05). Photodegradation has a significant impact upon vitamins break down: in fact, a statistically significant difference was noted between standards prepared in clear and amber glass autosampler vials soon after 7 days when kept at room temperature (*P* < 0.05; exact *P* = 0.017) and at +4°C (*P* < 0.05; exact *P* = 0.020); this difference increases over time, up to 30 days when the difference becomes statistically significant beyond 1% (*P* < 0.01; exact *P* = 0.0006). Ascorbic acid (vitamin C) and cyanocobalamin (B_12_) are the most sensitive to photodegradation.

Additionally, concentrations of vitamins were stable in processed (deproteinised) plasma samples and water-based controls for 24 hours when stored at +4°C prior to analysis [difference between peak area of vitamins in standard samples and peak area of vitamins in processed samples did not reach statistical significance when stored at +4°C for 24 hours, Student's *t*-test not significant, *P* > 0.05].

Three freeze-thaw cycles had an effect on the stability of vitamins (Student's *t*-test significant beyond 5%, exact *P* = 0.041). There is no statistically significant difference between freshly prepared, water-based standard solutions, and an aliquot of the same stored at −20°C after one cycle of freeze-thaw (*P* > 0.05), but the difference in peak area reaches statistical significance after two freeze-thaw cycles (*P* < 0.05; exact *P* = 0.042). In conclusion, standard solutions and trial samples can be stored at −20°C until the day of analysis for 30 days but they cannot be frozen again.

There was no clear evidence of carryover in any blank reagent samples.

Recovery tests were performed in triplicate by spiking blank plasma sample before deproteinisation (with 200 *μ*L pure acetonitrile) and solid-phase extraction with 20 ng/*μ*L of each vitamin. This concentration was selected as a compromise between the most possible plasma vitamins concentration to be found in real samples. Results were not significantly dissimilar to 100% [Student's *t*-test not significant, *P* > 0.05]. Recovery experiments were repeated at 75%, 50%, 25%, and 10% of the above vitamins concentrations. Recovery percentages ranged from 93% to 100% at all concentrations.

## 5. Application

Work with the participation of human subjects was conducted in accordance with the Declaration of Helsinki (1964). Twenty-four (24) abstinent alcohol-dependent males (average age: 51 ± 6 years old) undergoing alcohol rehabilitation agreed to have one blood and one urine sample taken to investigate their levels of water-soluble vitamins. All subjects who provided samples read, understood and signed a Participant Information Document. Ethical approval was obtained from both the University of Kent Ethics Committee (where the biochemical analyses were conducted) and from The Salvation Army Ethics Board (participants were residents in one Salvation Army Hostel near Swindon, UK).

Plasma samples were processed following the third methodology reported in Section 2 of this paper. Briefly, blood samples were withdrawn from participant using arm venipuncture, and plasma and serum fractions were immediately separated by centrifugation. Plasma fraction was used for the analysis of water-soluble vitamins. Plasma samples were treated with methanol: ethanol, 95 : 5, v/v and then processed through solid-phase extraction.

Results from plasma and urine analyses are reported in Tables 6 and 7, respectively. Unfortunately, it was not possible to separate and quantify vitamin B_3_ in plasma and urine samples.

## 6. Discussion

This paper reported the development and validation of a novel analytical method for the simultaneous detection and quantification of seven water-soluble vitamins in biological fluids (plasma and urine). The method has been shown to be robust and time-effective in the clinical routine practice. The application of this method to the analysis of water-soluble vitamins in plasma and urine samples of 24 abstinent alcohol-dependent males proved that the method is sensitive enough to detect low levels of analytes in complex matrices. This study provides an innovative approach to the simultaneous detection of seven water-soluble vitamins that has a wide range of application in clinical routine investigations.

## 7. Conclusions

The novel analytical method detailed in this paper has proved to be specific, robust, and time-efficient for the simultaneous detection and quantitation of water-soluble vitamins in complex biological matrices such as plasma and urine. This method can be used in routine clinical investigations where multivitamin analysis is required and low concentrations of vitamins are expected.

## Figures and Tables

**Table 1:** Column temperatures study. Experimental results (peak area mean ± RSD) obtained from six separate runs of a standard sample (aqueous mixture of 8 water-soluble vitamins) at 4 different column temperatures. The last row of the table reports run time in minutes. HPLC conditions used were those reported in the main text.

Vitamin	Peak area [mAU] ± R.S.D. measured at 280 nm at column temperature of 20°C	Peak area [mAU] ± R.S.D. measured at 280 nm at column temperature of 25°C	Peak area [mAU] ± R.S.D. measured at 280 nm at column temperature of 30°C	Peak area [mAU] ± R.S.D. measured at 280 nm at column temperature of 40°C
C	367.09 ± 0.03	333.94 ± 0.01	364.98 ± 0.04	143.68 ± 0.02
B_1_	168.78 ± 0.01	247.25 ± 0.03	169.66 ± 0.05	50.68 ± 0.06
B_2_	2065.76 ± 0.07	1681.83 ± 0.05	2138.75 ± 0.02	375.47 ± 0.03
B_3_	88.15 ± 0.03	89.61 ± 0.05	92.60 ± 0.02	44.51 ± 0.08
B_5_	51.05 ± 0.03	50.73 ± 0.08	60.13 ± 0.03	22.19 ± 0.09
B_6_	889.06 ± 0.02	924.11 ± 0.06	878.72 ± 0.08	191.70 ± 0.07
B_9_	181.25 ± 0.06	156.71 ± 0.05	205.21 ± 0.02	73.20 ± 0.03
B_12_	310.10 ± 0.02	532.52 ± 0.03	738.66 ± 0.05	574.27 ± 0.07

Total analysis time [mins]	25 min	25 min	35 min	45 min

**Table 2:** System suitability test results for the optimised HPLC method for determination of water-soluble vitamins in biological matrices (column temperature = 30°C; flow rate = 0.2 mL min^−1^). Mean peak areas of 5 injections of aqueous standard solution containing 20 ng mL^−1^ of each water-soluble vitamin.

Vitamin	Retention time (min)^a^ (%CV)^b^	Capacity factor (K′)	Selectivity (*α*)	Resolution (*R*_*s*_)	Tailing factor	plate count	Mean peak areas (mAU)^c^ (%CV)^b^
C	3.5 (0.9)	1.72	1.20	2.88	0.79	3111	365 (0.01)
B_1_	4.3 (0.7)	2.21	1.15	2.65	0.77	4943	170 (0.03)
B_3_	5.3 (1.1)	2.79	1.17	3.13	0.80	6056	93 (0.02)
B_6_ (PLP)	6.1 (0.99)	3.86	1.83	1.89	0.56	4255	879 (0.001)
B_5_	6.8 (0.29)	3.36	1.15	2.54	0.85	7050	60 (0.05)
B_9_	13 (0.07)	8.29	1.03	1.60	0.46	6735	205 (0.01)
B_12_	13.4 (0.6)	8.57	1.03	1.27	0.42	2329	740 (0.007)
B_2_	13.8 (0.2)	8.86	1.07	1.30	0.46	1096	2140 (0.001)

^a^The retention time of unretained peak is 1.4 min.

^b^%Coefficient of variation.

^c^milli Arbitrary Unit.

**Table 3:** Method optimisation results. Results from the sample preparation method optimisation for vitamins spiked in artificial plasma samples processed using each of the three methods mentioned above. Results are reported as mean of peak area ± RSD (*n* = 3). Numbers in parenthesis indicate wavelength used for quantitation.

Vitamins (nm)	Peak area [mAU]	% RSD
200 *μ*L pure acetonitrile + solid phase extraction		
C (230)	150.92	2.6
B_1_ (270)	62.60	1.7
B_2_ (265)	366.16	10.3
B_5_ (266)	27.14	0.4
B_6_ (257)	110.28	2.4
B_9_ (280)	69.13	2.0
B_12_ (230)	179.13	5.2
L/L extraction_aqueous phase		
C (230)	197.91	4.7
B_1_ (270)	64.10	2.9
B_2_ (265)	216.31	6.0
B_5_ (266)	14.83	0.2
B_6_ (257)	145.07	9.4
B_9_ (280)	112.93	0.9
B_12_ (230)	132.36	2.6
600 *μ*L ethanol : methanol (95 : 5) + solid-phase extraction		
C (230)	169.14	2.8
B_1_ (270)	87.75	2.3
B_2_ (265)	302.29	1.9
B_5_ (266)	45.52	0.7
B_6_ (257)	60.97	1.0
B_9_ (280)	285.33	5.4
B_12_ (230)	111.74	1.0

**Table 4:** Regression parameters of analysed vitamins. Regression parameters (regression equation [±SD of slope and intercept] and regression coefficient) and detection limits (signal-to-noise ratio = 5 and injection volume = 3 *μ*L).

Vitamin	Limit of detection [ng *μ*L^−1^] at given UV (*λ*, [nm])	Regression line	Regression coefficient
C	0.5 ng *μ*L^−1^ (230 nm)	*y* = 0.657(±0.003)*x* + 127.1(±0.002)	*R* ^2^ = 0.994
B_1_	1 ng *μ*L^−1^ (270 nm)	*y* = 8.789(±0.002)*x* + 16.31(±0.004)	*R* ^2^ = 0.996
B_2_	0.5 ng *μ*L^−1^ (265 nm)	*y* = 301.7(±0.005)*x* + 467.3(±0.002)	*R* ^2^ = 0.996
B_5_	1 ng *μ*L^−1^ (266 nm)	*y* = 1.052(±0.008)*x* + 66.43(±0.003)	*R* ^2^ = 0.979
B_6_ (PLP)	0.5 ng *μ*L^−1^ (257 nm)	*y* = 7.451(±0.003)*x* + 12.81(±0.004)	*R* ^2^ = 0.997
B_9_	1 ng *μ*L^−1^ (280 nm)	*y* = 246.1(±0.002)*x* + 54.31(±0.005)	*R* ^2^ = 0.999
B_12_	2 ng *μ*L^−1^ (230 nm)	*y* = 132.9(±0.007)*x* + 209.6(±0.008)	*R* ^2^ = 0.995

**Table 5:** Retention times and peak areas of analysed vitamins. Relative standard deviations (%RSD) of retention times and peak areas for 7 water-soluble vitamins spiked at 20 ng *μ*L^−1^ in artificial plasma obtained in the analysis for repeatability and reproducibility.

Vitamin	Repeatability^a^		Reproducibility^b^	
	Retention time %RSD	Area %RSD	Retention time %RSD	Area %RSD
C	0.56%	2.23%	1.97%	4.56%
B_1_	0.99%	3.65%	2.76%	6.89%
B_2_	0.13%	1.87%	0.89%	3.3%
B_5_	0.90%	2.25%	2.75%	4.1%
PLP	0.46%	2.22%	1.93%	3.77%
B_9_	0.18%	2.69%	1.2%	2.98%
B_12_	0.67%	2.98%	2.37%	6.07%

**Table 6:** Mean concentration of individual water-soluble vitamins (±SEM) in plasma samples obtained from 24 study participants. Elevate variability in individuals' vitamin levels may explain high S.E.M.s in the case of B_1_, B_5_ and B_6_.

	C (*μ*mol L^−1^)	B_1_ (nmol L^−1^)	B_2_ (nmol L^−1^)	B_5_ (nmol L^−1^)	B_6_ (nmol L^−1^)	B_9_ (nmol L^−1^)	B_12_ (nmol L^−1^)
Mean (*n* = 24)	44.7	51.3	15.6	32.4	73.3	8.5	5.7
S.E.M	16.3	27.6	5.5	32.7	37.9	4.3	1.7
Ref. value [1, 2]	24–84	9–44	6.2–39	N/A	7–52	3.1–18	1.9–3.5

Ref. values: reference intervals obtained from Malmauret et al. 2002 [1] and from Talwar et al. [2].

**Table 7:** Average concentration of individual water-soluble vitamins (±SEM) in urine samples obtained from study participants.

	C	B_1_	B_2_	B_5_	B_6_	B_9_	B_12_
Average concentration (*n* = 24), mg dL^−1^	0.04	0.15	0.01	0.14	0.23	0.02	0.02
S.E.M. (mg dL^−1^)	0.02	0.03	0.00	0.01	0.09	0.01	0.01
Av. Creat. (mg dL^−1^)	96.86	96.86	96.86	96.86	96.86	96.86	96.86
Vitamin/creatinine ratio	0.00038	0.00153	0.00012	0.00143	0.00237	0.00020	0.00025
